# Residual-Based Multi-Stage Deep Learning Framework for Computer-Aided Alzheimer’s Disease Detection

**DOI:** 10.3390/jimaging10060141

**Published:** 2024-06-11

**Authors:** Najmul Hassan, Abu Saleh Musa Miah, Jungpil Shin

**Affiliations:** School of Computer Science and Engineering, The University of Aizu, Aizuwakamatsu 965-8580, Japan; musa@u-aizu.ac.jp

**Keywords:** Alzheimer’s disease, residual network, CNN, machine learning, Random Forest

## Abstract

Alzheimer’s Disease (AD) poses a significant health risk globally, particularly among the elderly population. Recent studies underscore its prevalence, with over 50% of elderly Japanese facing a lifetime risk of dementia, primarily attributed to AD. As the most prevalent form of dementia, AD gradually erodes brain cells, leading to severe neurological decline. In this scenario, it is important to develop an automatic AD-detection system, and many researchers have been working to develop an AD-detection system by taking advantage of the advancement of deep learning (DL) techniques, which have shown promising results in various domains, including medical image analysis. However, existing approaches for AD detection often suffer from limited performance due to the complexities associated with training hierarchical convolutional neural networks (CNNs). In this paper, we introduce a novel multi-stage deep neural network architecture based on residual functions to address the limitations of existing AD-detection approaches. Inspired by the success of residual networks (ResNets) in image-classification tasks, our proposed system comprises five stages, each explicitly formulated to enhance feature effectiveness while maintaining model depth. Following feature extraction, a deep learning-based feature-selection module is applied to mitigate overfitting, incorporating batch normalization, dropout and fully connected layers. Subsequently, machine learning (ML)-based classification algorithms, including Support Vector Machines (SVM), Random Forest (RF) and SoftMax, are employed for classification tasks. Comprehensive evaluations conducted on three benchmark datasets, namely ADNI1: Complete 1Yr 1.5T, MIRAID and OASIS Kaggle, demonstrate the efficacy of our proposed model. Impressively, our model achieves accuracy rates of 99.47%, 99.10% and 99.70% for ADNI1: Complete 1Yr 1.5T, MIRAID and OASIS datasets, respectively, outperforming existing systems in binary class problems. Our proposed model represents a significant advancement in the AD-analysis domain.

## 1. Introduction

Alzheimer’s, a neurological disease, is the leading cause of the common type of dementia and chronic disease that destroys neurons [1,2]. Recent research in Japan’s Hisayama region found that over 50% of elderly individuals face a lifetime risk of dementia, with more than half of these cases attributed to AD [3]. According to the World Health Organization, many people will be affected by AD in the next year, and the number is projected to exceed 152 million worldwide by 2050, surpassing global cancer patient statistics [4]. AD is a major neurocognitive impairment affecting memory, abstract thinking, judgment, behavior and emotion [5]. In addition, AD, as it progresses, can significantly impact an individual life—the early symptoms of AD manifest as forgetfulness regarding recent events. However, AD may lead to difficulty performing daily activities independently [6,7,8]. In addition, AD negatively affects behavior, social interaction and cognitive skills. In patients in the early stage of AD, communication challenges result in difficulty answering questions or reacting to their surroundings. Although AD mainly affects individuals aged 65 and older, it is not exclusively a disease of old age, as young-onset cases also occur frequently [9]. There is no standardized test that definitively indicates you have AD or no Alzheimer’s mild cognitive impairment (MCI) [10]. Assessments of patients involve medical history, physical condition examination, and various diagnostic tests, including Nero’s logical examination such as muscle tone and reflex coordination [11]. Furthermore, imaging modality techniques such as MRI and computed tomography (CT) play an important role in the diagnostic process for AD [12]. AD is a prevalent condition, and timely and early/accurate diagnosis plays a very crucial role in improving patients’ quality of life. According to recent studies, various efficient ML-based algorithms and DL-based models have been developed to determine the accuracy of AD prediction from MRI. In recent years, researchers worldwide have been developing numerous ML-based [13] and DL-based [14,15] algorithms to identify and categorize AD. While some have succeeded with DL-based algorithms, there is still a gap for improvement. Various DL models, like hybrid CNN, combining slice selection and histogram stretching, and others incorporating skull stripping techniques [16], have been introduced [17,18,19,20]. Among the available datasets, the Alzheimer’s Disease Neuroimaging Initiative (ADNI) dataset stands out as one of the most challenging. Researchers have been striving to develop AD-recognition systems using DL technologies to enhance performance accuracy and efficiency with the ADNI dataset. For instance, Kamal et al. achieved 82.00% accuracy with a CNN-SVC system and evaluated the Kaggle dataset [21]. Similarly, other researchers reported high accuracies with models like DEMNET [22] and CNN [23]. However, these models may not meet satisfactory performance accuracy, computation complexity and efficiency levels.

Given these circumstances, there is an urgent need to develop a more efficient Alzheimer’s disease-detection module to overcome the challenges of performance accuracy and efficiency. More recently, Saeed et al. proposed a pre-trained DL-based model, ResNet50, which was used to extract features, and then the ML-based algorithm SVM, RF, and DL-based SoftMax approach were applied. After evaluating the experiment of their model with the ADNI dataset, they achieved 85.70%, 92.00% and 99.00% accuracy with RF, SVM and SoftMax classifier, respectively, for the binary class classification [24]. They also evaluated the MIRIAD dataset and reported 84.80%, 90.00% and 96.00% accuracy, respectively. The main drawback of the existing system is it uses hierarchical CNN architecture, and deeper neural networks are more difficult to train [25]. It also causes low-performance accuracy because of optimization issues and a lack of feature effectiveness. More recently, Arafa et al. applied a hybrid model by integrating VGG16 and transfer learning, where they reported 97.44% accuracy with the ADNI Kaggle version dataset for binary classification [26]. Loddo et al. employed a strategy of deep learning approaches, including InceptionNet, AlexNet and ResNet [27] for AD diagnosis. They reported 98.51% averaged accuracy for the binary class classification for four different AD datasets.

The main drawback of these [24,26,27] works is that their performance accuracy is not high enough to reach a satisfactory level, and their computational complexity is high. To overcome the problem, we proposed a residual function-based [25] multi-stage deep neural network to increase the feature effectiveness aiming to increase the performance accuracy. Our contributions to the proposed work are given below:

- **Novelty of the Proposed System:** We propose here five stages of the deep learning module where each stage is explicitly reformulated with the other layers’ known case residual functions with reference to the layer inputs. Using five stages, we aim to extract hierarchical features by keeping the depth of the proposed module.
- **Feature Selection and Classification:** After extracting the effective feature, we employed a deep learning-based future-selection module, which is constructed with the batch normalization layer, dropout layer and fully connected layer to protect the overfitting of the proposed system. Then, we used two ML-based and one DL-based classification algorithms, namely SVM, RF and SoftMax.
- **Comprehensive Evaluation:** To evaluate the proposed model, we used three benchmark datasets, namely ADNI1: Complete 1Yr 1.5T, MIRAID (Minimal Interval Resonance Imaging in Alzheimer’s Disease) and OASIS dataset. The proposed model achieved 99.47%, 99.10% and 99.70% accuracy for ADNI1: Complete 1Yr 1.5T, MIRAID and OASIS datasets, respectively. In each case, our proposed model performed better than the existing systems for the binary class problems. Because of its novelty, this work will be considered a new invention in the domain of Alzheimer’s-recognition research.

The structure of the paper is as follows: Section 2 presents a review of existing approaches for AD detection, while Section 3 presents the datasets and a description with preprocessing steps; Section 4 presents the proposed methodology and explains three different classifiers. Section 5 provides the experimental evaluation results, including the parameter values and comparison with recent models. Finally, in Section 6 we present the conclusion and lay out future work.

## 2. Related Work

Many researchers have developed systems for diagnosing Alzheimer’s disease using MRI images. Researchers have utilized various datasets, tools and techniques to make a system for AD classification. Recently, researchers have focused on two main approaches for training and classifying models, conventional learning and deep learning, as they proved excellent in other domains [28,29,30]. Recent studies like [24,31,32,33,34] used conventional learning for AD classification, while studies like [5,35,36,37,38] employed deep learning techniques.

Al-Adhaileh et al. [36] employed two pre-trained CNN-based models (AlexNet, ResNet50) to find the optimal model for AD classification. They used the Kaggle ADNI version dataset to train the models and perform the four-class classification. They split the dataset into 80% for training and 20% for testing. They train the first model, AlexNet, which consists of 34 layers and five max-pooling with size 4×4, whereas the second model, train ResNet50, consists of 177 layers and five max-poling layers with size (5×3). Finally, they employed the ReLu activation and SoftMax functions in the final layers of both models to classify the four classes. The AlexNet model outperformed the ResNet50 model, with a reported accuracy of 94.5% and 58.07%, respectively. They demonstrate that the AlexNet model is more accurate than ResNet50. Antony et al. [39] suggest two pre-trained models, VGG16 and VGG19, to find the best model for AD classification. These two models were trained on the ADNI dataset comprising 780 images. In the pre-processing stage, they perform data augmentation before training the model. Finally, they used the Sigmoid activation function as a classifier on the VGG16 model, while they used the SoftMax activation function as a classifier on the VGG19 model for binary class classification, respectively. The reported accuracy is not good for both models; VGG16 attained 81.00% and VGG19 attained 84.00%. Raza et al. [40] employed a pre-trained model as their base and then used the CNN-based transfer learning approach to fine-tune the model for AD classification. Specifically, they utilized the images segmented by the brain’s grey matter. The reported accuracy is 97.84%, but the model is insufficient regarding the computation cost. Similarly, Mehmood et al. [41] utilized layer-wise transfer learning techniques with a pre-trained deep-learning model (specifically the VGG architecture) that is fine-tuned for AD classification. They extracted the grey matter from the MRI scan attained from the ADNI database. This grey matter was utilized to fine-tune the pre-train model while preserving features from the image net database and achieved an accuracy of 98.73% between AD and CN. Rallabandi et al. [42] developed an algorithm based on conventional learning for early diagnoses of AD and binary class classification between AD and MCI. They used the ADNI dataset of 1167 whole-brain MRI subjects, including NC, early MCI, late MCI and AD. They calculated 68 features using the Free Surfer analysis of each MRI scan and then utilized these features to build the model. In addition, they tested the scan to classify four groups utilizing various ML techniques such as nonlinear SVM (RBF kernel), linear SVM, naive Bayesian, KNN, decision tree and RF. The best classification performance was achieved by a nonlinear SVM with (RBF kernel) a classifier and had an accuracy of 75.00%.

The Kaggle ADNI version dataset was used by Chabib et al. [38] to employ curvelet transform (CT) based on the CNN model to determine early-stage AD detection. They first used CT for pre-processing, then a CNN model was trained to utilize it for new image representation. The experiments were evaluated to train the model for both binary and multi-class classification and reported an accuracy of 98.71% and 99.05%, respectively. Liu et al. [43] employ the OASIS dataset and developed a CNN-based model that consists of three multi-convolutional layers, three pooling layers, and an FC layer with SoftMax as the last layer of the model. Furthermore, they used two train networks, AlexNet and GoogleNet, for transfer learning to improve classification accuracy. The reported accuracy of 78.02%, 91.40%, and 93.02% is achieved by the CNN, AlexNet, and GoogleNet models, respectively. Abdul et al. [35] employed an end-to-end CNN-based model for an AD-detection system. They applied the augmentation techniques on the training dataset and performed three experiments to show the effectiveness of the model. Furthermore, they used the ADNI dataset for the binary classification of AD vs CN and reported an accuracy of 95.5%. Savas et al. [44] used the ADNI dataset and implemented various CNN models to classify 2182 image objects. They compared the performance of the model with 29 pre-trained models. In addition, they used data-augmentation techniques for pre-processing to clean the data and divide the images. During the training and testing stage for early AD detection, EfficientNetB0, EfficientNetB2, and EfficientNetB3 achieved an accuracy of 92.20%, 94.42%, and 97.28%, respectively. Meng et al. [32] employ a model for AD diagnosis to perform the voxel-based morphometry (VBM) data obtained from MRI scans consisting of 1426 MRIs. They extracted the features based on a random survey Support Vector Machine (RS-SVM) approach. This method combines image gene and pathway levels to analyze and classify AD. The RS-SVM method was compared with other MLS methods like (linear regression, Lasso, partial least squares, and SVM) and achieved an accuracy of 91.00% for AD vs HC. In a recent study, Kong et al. [5] employ 3DCNN to extract features from multi-model data such as structural magnetic resonance imaging (MRI) and positron emission tomography (PET) for AD detection. They attained an accuracy of 93.21% for two classes. Kaplan et al. [45] proposed the AD-detection system for the binary class by utilizing the Kaggle ADNI version dataset. They extracted features using the feed-forward local phase quantization network (LPQNeT), and then these features were fed into conventional classifiers like SVM to achieve an accuracy of 89.84%.

Zang et al. [37] employ a deep belief network (DBN) based on a multi-task learning (MLT) algorithm using the ADNI dataset and performing various tasks for AD classification and reported accuracy of 98.60% for binary classes.

Alseed et al. [24] suggest a pre-trained CNN DL-based model (ResNet50) for automatic feature extraction from MRI images. They used a data-augmentation technique to handle the data imbalance problem. After feature extraction, they utilized three classifiers (SVM, RF, and SotfMax) and reported accuracy of 92.00%, 85.70%, and 99.00%, respectively. Table 1 summarizes the reviewed recent studies and provides a comparison based on the following key aspects: methods, dataset, image type, classification, and accuracy. According to the information in the table, recent studies have faced challenges such as a lack of effective features, high computational complexity, and performance accuracy. Therefore, the main objective of this study is to implement an analysis of CNN feature extraction for the automatic classification of AD and propose a residual-based multi-stage deep learning-enhanced model with SVM, RF, and a SoftMax classifier.

## 3. Dataset

We used three publically available datasets, namely ADNI1: Complete 1Yr 1.5T (https://adni.loni.usc.edu/ accessed on 1 December 2023), OASIS [50,51], and MIRIAD [52], to evaluate our proposed Residual-Based Multi-Stage Deep Learning (RBMSDL) model. Table 2 depicts the number of images of each class, and the description of each dataset is given in the following subsections.

### 3.1. MIRIAD Dataset

**Figure 1:**
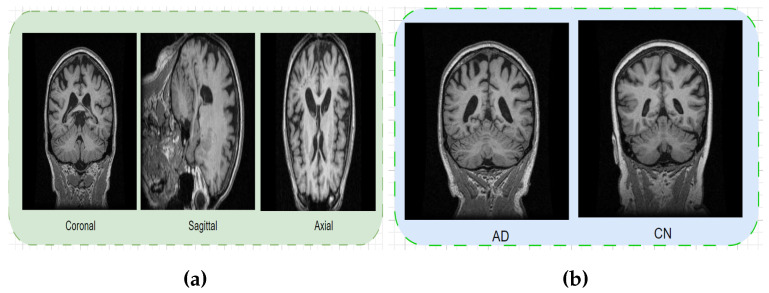
Visual information of brain MRIs: (**a**) Different view of MRI imaging plans, (**b**) Normal brains and affected by AD.

The MIRIAD dataset [52] is publicly available in the form of NIFT1 and the file extension is .nii using different tools to provide the details of the brain MRI images and analysis of the anatomy of the three planes axial, sagittal, and coronal shown in Figure 1a. This dataset has information on 46 people with Alzheimer’s disease (AD) and 23 people with control normal (CN). We collected multiple scans from each person over different periods, from 2 weeks to 2 years. The goal of the study was to see if MRI scans could be used to measure the effects of treatments for AD in clinical trials. In total, we gathered 708 MRI scans from both CN and AD cases. We used a specific type of MRI called 3D T1-weighted MRI, which uses the IR-FSPRG sequence to capture detailed brain images. The image characteristics are the field of view 24 cm, matrix size (256×256×124) slice thickness 1.5 mm (coronal position) TR 15 ms, epoch time (TE) 5.4 ms, flip angle 15 degrees, inversion time 650 ms. Note that the dataset did not specify the degree of AD. In our experiments, we first convert the .nii files into MRI images using the med2image (https://github.com/FNNDSC/med2image accessed on 1 February 2024) techniques and selected axial planes [52] for AD and CN. Figure 1b depicts the visual comparison between AD and CN.

### 3.2. OASIS Kaggle Version Dataset

We collected a dataset from the Kaggle OASIS [50], which consists of 80,000 brain MRI images with patients who suffer from diseases or not. After checking the dataset contains four classes of MRI images, namely non-demented (NOD), moderate demented (MOD), mild demented (MID), and very mild demented (VMD). The first class is NOD a person who has no AD, the second class is MOD a person who has early AD, the third is the MID a person who has a more severe case than the MOD and the final class is VMD a person who has a high degree of AD. Among these classes, the NOD class contains 67.2 k samples, while the VMD, MID, and MOD classes contain 13.7 k, 5002, and 488 images, respectively. It is worth noting that the dataset is imbalanced. We chose two classes, namely NOD and VMD, which consist of a total of 6400 MRI images. Table 2 provides a detailed dataset description, while Figure 1b shows the visual results.

### 3.3. ADNI1: Complete 1Yr 1.5T

We collected the dataset in the form of MRI’s ADNI1: Complete 1Yr 1.5T from the publicly available (https://adni.loni.usc.edu/ accessed on 1 December 2023). The ADNI aims to identify biomarkers for the disease to perform early diagnosis of AD. In addition, the aim was to gain a better understanding of the clinical treatment of the pathophysiology of AD. The full description of the dataset contains a total of 2294 MRI scans that consists of 639 subjects labelled AD, CN, and MCI. Then, we find the MRI scan of the ADNI dataset in the form of NIFTI files with extension (.nii). We selected 3000 brain MRI axial AD images and 3000 brain MRI axial CN images and fixed the size of each image 256×256 for further processing.

## 4. Proposed Methodology

**Figure 2:**
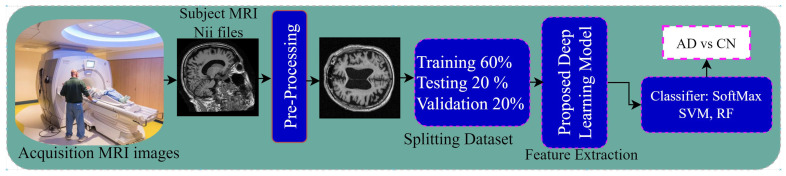
Outline of the proposed model.

**Figure 3:**
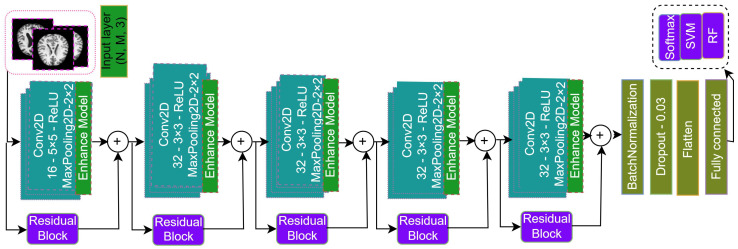
Proposed Residual-Based Multi-Stage Deep Learning (RBMSDL) model architecture.

**Figure 4:**
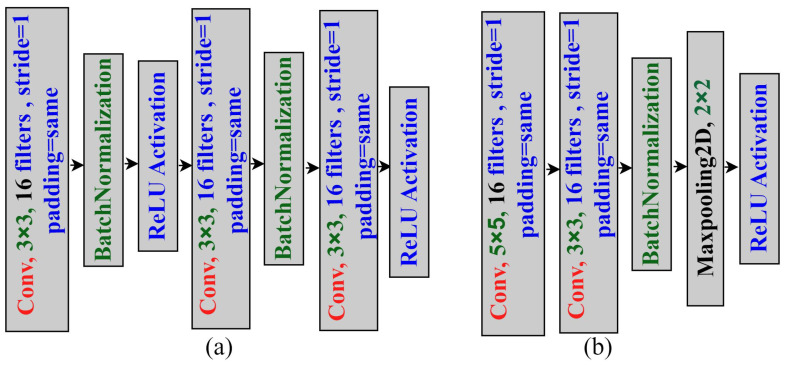
(**a**) Enhanced module, (**b**) residual module.

The workflow architecture of the proposed methodology is demonstrated in Figure 2 and Figure 3. Our primary objective is to design an early AD-diagnosis approach. The proposed methodology for the RBMSDL (Residual Block Multi-Stage Deep Learning) model involves several key steps aimed at extracting relevant features from MRI images and improving the classification of brain MRI images for early AD diagnosis. According to Figure 2 and Figure 3, our methodology comprises three main components:

- **Novel Multi-Stage Deep Neural Network Architecture:** We proposed a novel Residual-Based Multi-Stage Deep Learning (RBMSDL) approach for AD detection as demonstrated in Figure 3. This architecture consists of a five-stage block, each block explicitly formulated with a convolutional block and known residual module to enhance feature effectiveness while maintaining model depth. In the procedure, we implemented five stages of residual blocks integrated with a CNN model to extract relevant features from MRI images. By reformulating each stage with reference to layer inputs, we aim to extract hierarchical features that capture the underlying complexity of AD. The main task of each residual module is to enable effective training in the deep network to facilitate gradient flow, introduce non-linearity, and learn the hierarchical representation of features from input MRI images. In the first stage block, we fed the preprocessed MRI image dataset into the convolutional module and residual module, where the convolutional module produced the high-depth spatial feature by integrating the convolutional layer and max pooling layer with the enhancement module. The residual module produced the low-depth spatial feature aiming to recover the information loss during the convolutional block. The enhanced module and the residual module are demonstrated in Figure 4a,b, respectively. Then, element-wise addition between the convolutional module output and residual block output is fed into the second stage block. Sequentially, we fed the output of the second stage block into the third stage block, and we used five stage blocks here to produce the hierarchical feature using multistage integration of the convolution and residual block. After the fifth stage, we obtained the final feature. Moreover, in addition to the convolutional module, the residual modules are important in addressing the vanishing gradient problem that occurs in deep neural networks (DNNs) [25] during training. The skip connections (or identity mappings) within each residual module facilitate the flow of gradients during backpropagation. This helps mitigate the degradation problem and enables the training of deeper networks to be more effective.
- **Feature Selection**: Following effective feature extraction, we implement a deep learning-based feature-selection module to mitigate overfitting and ensure the robustness of our proposed system. This module incorporates batch normalization, dropout, and fully connected layers to optimize feature selection.
- **Classification Techniques:** We fed the reduced feature vector into the classification module. In the study, we utilized three classification approaches, SVM, RF, and the SoftMax approach. The objective of using a three classifier is to obtain robust classification performance. To evaluate the performance of our proposed RBMSDL model, we conduct comprehensive evaluations using three benchmark datasets: ADNI1: Complete 1Yr 1.5T, OASIS Kaggle version, and MIRAID. We measure the accuracy of our model on each dataset, specifically focusing on its performance in binary class problems. Our evaluation procedures aim to demonstrate the superior performance of the proposed RBMSDL model compared to existing systems, thereby validating its effectiveness in AD detection.

Overall, our methodology integrates innovative approaches to feature extraction, selection, and classification, culminating in a robust and accurate AD-detection system. Through rigorous evaluation of diverse datasets, we aim to establish the efficacy of our proposed RBMSDL model and contribute to advancements in Alzheimer’s recognition research.

### 4.1. Preprocessing and Model Initialization

Firstly, the dataset collection is mentioned in Section 3. In the second dataset pre-processing steps, we check the image size and resize it for our model. The pre-processing steps for ADNI-1 Complete 1yr 1.5T and MIRIAD datasets are discussed here. The dataset’s original format is NIFTI, with the file extension (.nii). We were preparing to use various tools before using them. We use the FSL [53] tool to analyze brain images to obtain MRI, fMRI, and DTI to view the different angles of brain images. The operation, executed using FSL performs several preliminary procedures such as reorientation, neck portion reduction, skull stripping, preliminary linear registration, and non-linear registration. The pre-processing phase of the dataset aims to transform it into the optimal representation needed to input requirements. First, we convert the .nii files of each class into MRI images using the full procedure pre-processing step available at the following link: (https://github.com/najm-h/Alzheimer/ accessed on 1 February 2024). Next, we extracted the MRI images into three planes, axial, sagittal, and coronal, using the med2image (https://github.com/FNNDSC/med2image accessed on 1 February 2024) techniques, as depicted in Figure 1a. Figure 1 demonstrates the preprocessing output images. Next, we divided the datasets into 60%, 20%, and 20% training, validation, and testing, respectively. Finally, we extracted the features and trained and tested our proposed models.

### 4.2. CNN with Residual Blocks

The architecture of the proposed RBMSDL model consists of five convolution blocks with residual connections, maximum pooling layers, and FC layers. The CNN with residual block combination is the power of convolutional layers (CLs) to learn both low- and high-level features. Each block consisted of the enhanced module and residual block module, which were beside the initial one. Figure 4a demonstrates the details of the enhance module, and Figure 4b demonstrates the details of the residual module. The residual block network (ResNet) is known as a deep neural network (DNN) and is used to overcome the degradation problem observed in DNN [25]. The degradation problem refers to the phenomenon where adding more layers to a neural network leads to higher training errors, contrary to the expectation that a deeper neural model should perform as well as a shallower one. We added the residual or skip connections within the network architecture to address the degradation issues. The residual block is the main fundamental building block in deep neural networks. Each residual block consists of 2 CLs followed by a maximum pooling layer, batch normalization, and ReLu activation function. The main concept of the residual block is the residual connection, where the output CL is added to the original input tensor. This proposed approach allows the network to learn the residual function, which performs capture between the desired mapping and the current approximation of the network. Introducing the residual connection enhances the training efficiency and improves the gradient flow of the deep networks.

Meanwhile, convolution blocks with residual connections and a maximum pooling layer are utilized for effective feature extraction, while FC layers are used for the AD classification stage. Additionally, for the best classification, we use three types of classifiers, (SoftMax, SVM, and RF) to investigate which one has the best performance in terms of accuracy and is computationally efficient. We explain each step in more detail in the following sections.

#### 4.2.1. Convolutional Layer

The convolution layer (CL) is the main building block of deep neural networks, and performs feature extraction while producing the output set of 2D matrices known as feature maps. Each CL performs effective feature extraction by applying a fixed number of filters to the input image. In the proposed RBMSDL model, we used the filter of size 5×5 and 3×3. Each filter learns to detect and recognize patterns like colors, edges, shapes, and textures during the training process.

#### 4.2.2. Pooling Layer

The pooling layer is typically fixed after the CL in the CNN architectures. The pooling layers aim to downsample the feature map produced by CL. The max pooling in particular operates to reduce feature maps by selecting the max value with the small regions in the image, usually 2×2. The max pooling is performed based on the partition of the image into nonoverlapping (2×2) regions. However, the maximum value is selected for each region while max pooling is performed to prevent overfitting by abstracting the image’s visual representation. Moreover, it reduces the number of parameters and minimizes the computational cost. Furthermore, like the max pooling layer, average pooling also operates on the 2×2 region of the images. However, this layer computes the average value instead of the maximum values. Finally, the max pooling layer emphasizes specific features, while the average pooling layer provides a smoother representation.

#### 4.2.3. Batch Normalization

The primary goal of batch normalization [54] aims to normalize the output of CLs by setting the batch mean to 0 and variance to 1. This approach stabilizes the training process and allows for the utilization of a higher learning rate. Moreover, it mitigated the vanishing gradient problem during the backpropagation process. Additionally, the DL model with batch normalization layers is more robust to variation in weight initialization; it reduces the sensitivity to improper initial weight.

#### 4.2.4. Dropout Layer

To prevent the overfitting of the system, we used the dropout layer [55], which mainly worked by deactivating neurons during the training period. This layer also enhances model generalization by promoting robustness to noise and variations in the input data. This regularization technique encourages the network to learn more robust features by preventing co-adaptation of neurons, thereby improving its ability to generalize to unseen data. The dropout rate parameter regulates the probability and extent of neuron exclusion. Importantly, neuron removal occurs only during the training process. Crucially, neuron removal exclusively takes place throughout the training phase. This layer acts as an ensemble method within the network, effectively training multiple subnetworks simultaneously and combining their predictions. This ensemble effect contributes to further regularization and enhances the model’s predictive performance.

#### 4.2.5. Fully Connected Layer

In addition to serving as a classifier, the fully connected (FC) layer plays a crucial role in feature extraction and representation learning within the proposed RBMSDL model for Alzheimer’s disease recognition. By connecting multiple layers of the network, the FC layer facilitates the extraction of high-level features from the input data, enabling the model to capture complex patterns and relationships relevant to disease diagnosis. Furthermore, the FC layer contributes to the interpretability of the model by providing insights into the learned representations, thereby aiding in the understanding of disease-related biomarkers and features. Additionally, the use of the SoftMax activation function after the FC layer not only normalizes the output results but also enables probabilistic interpretation of the model’s predictions, allowing for confidence estimation in the classification outcomes.

### 4.3. Classification Module

In this study, we extract effective features and input them into the classification module. We utilize SoftMax, SVM, and RF classifiers to achieve optimal classification results. Each classifier offers unique strengths: SoftMax provides probabilistic outputs for intuitive interpretation, SVM handles high-dimensional data with non-linear decision boundaries effectively, and RF excels in robustly handling large datasets with high dimensionality. By employing this diverse set of classifiers, our classification module ensures robust and accurate classification results across a variety of data types and complexities.

#### 4.3.1. SoftMax Classifier

In CNN architectures, the SoftMax function serves as the final layer, providing several key advantages essential for robust label data classification. Firstly, it computes the probability distribution over multiple classes, assigning a probability value between 0 and 1 to each class, thereby offering insights into the model’s confidence level for each prediction. This probabilistic interpretation enhances the model’s interpretability and facilitates decision-making processes, especially in medical applications like Alzheimer’s disease diagnosis [24]. Moreover, the SoftMax function ensures that the sum of probabilities across all classes equals one, guaranteeing a normalized output suitable for classification tasks. This normalization property enhances the stability and reliability of the classification process, particularly when dealing with imbalanced datasets or noisy input. Additionally, by transforming the model’s raw output into interpretable probabilities, the SoftMax function enables meaningful comparisons and assessments of model performance, facilitating model evaluation and refinement [56]. Mathematically, the SoftMax function is expressed as follows:

(1) $$f{\left(y\right)}_{i}=\frac{e^{y_{j}}}{\sum_{k=1}^{K}e^{y_{k}}}\;\mathrm{for}\;j=1,\ldots ,K$$

where *k* denotes the dimension of the random value of (*y*), and the SoftMax function f(y) output value is converted into meaningful probabilities between 0 and 1.

#### 4.3.2. Support Vector Machine Classifier

SVM is a well-known supervised ML-based algorithm that can be utilized for different tasks, including classification and regression, and we utilize it as a binary image classifier. The main goal of SVM is to find an optimal hyperplane that aims to divide N-dimensional space that can separate data points belonging to different classes based on the hyperplane to maximize the margin as much as possible. In addition, the dimensions of the hyperplane depend on the number of features. For 2-dimensional features (two features), the hyperplane is a line; for 3-dimensional features, the hyperplane becomes a 2-dimensional plane; and for higher dimensions, it generates subspace. Finally, we replace the FC layers with an SVM classifier and experiment with the linear kernel in this research. The linear kernel commonly operates in the input feature spaces, performs well, and creates the decision boundary linearly. Specifically, the linear kernel computes the dot product between input vectors (x,y) and effectively linear relationship.

#### 4.3.3. Random Forest Classifier

RF stands out as a potent algorithm specifically designed to minimize prediction variance. It goes beyond bagging, a similar ensemble method, by constructing a vast ensemble of decor-related decision trees and subsequently averaging their predictions. This approach addresses the main goal of bagging, which is to average out minor noise while employing an almost unbiased model to reduce variance effectively. The utilization of trees is particularly apt for bagging due to their ability to capture complex interactions within the data. By aggregating the predictions of numerous trees, Random Forest effectively reduces the risk of overfitting while maintaining high predictive accuracy [57]. It is used for both classification and regression. In the case of the classification problem, each tree contributes a class vote, and then the final classification is based on a majority vote. In the case of regression, the prediction of each tree at a target point is averaged. In this study, we used RF for the classification and adjusted the hyperparameter, such as the number of estimators, which is a crucial parameter, while the default value is 100, and it can be changed from 1 to 100. The RF often performs comparably to hosting the methods, but they are simple to fine-tune. Consequently, they have gained popularity and are widely implemented in various packages.

## 5. Experimental Evalution

We evaluated the proposed RBMSDL-enhanced model using the publicly available datasets ADNI1: Complete 1Yr 1.5T (https://adni.loni.usc.edu/ acessed on 1 December 2023), MIRIAD [52] and OASIS Kaggle version [50] to achieve good results in terms of accuracy.

### 5.1. Environmental Setting for Experiments

To evaluate the proposed model, we divided the datasets into 60%, 20%, and 20% training, validation, and testing, respectively. Finally, we extracted the features and trained and tested our proposed RBMSDL models. It is crucial to identify the most appropriate approach for designing the diagnostic system for AD to achieve superior accuracy. Our experiments were implemented across various hardware and software environments. This experiment was carried out in a Python environment, Python packages, and DL libraries like TensorFlow, OpenCV Scikit-learn, and Numpy. We implemented the system on a GPU PC with an Intel^®^ Core^™^ i9 13900K CPU, 64 GB RAM, Ubuntu and an NVIDIA^®^ Geforce RTX^™^ 4090. We used the optimizer SGD, learning rate 0.001, 50 epochs for the model run, and for the batch size we configured it to 8. For the efficacy and the performance of our proposed RBMSDL-enhanced model, we evaluated the following metrics: accuracy, F1-score, recall (REC) or sensitivity, and precision (PRC).

(2) $$\mathrm{Accuracy}=\frac{TP+TN}{TP+TN+FP+FN}$$

(3) $$F1\text{-}\mathrm{Score}=\frac{2TP}{2TP+FP+FN}$$

(4) $$\mathrm{REC}=\frac{TP}{TP+FN}$$

(5) $$\mathrm{PRC}=\frac{TP}{TP+FP}$$

where true positive (TP) indicates that the actual class label is an AD subject, while the system is also predicted as an AD subject. False positive (FP) means that the actual class label is not an AD subject, but the system has predicted the AD subject. True negative (TN) indicates that the actual class label is not an AD subject, but the system predicted it is not an AD subject. False negative (FN) means that the actual class label is an AD subject, but the system predicted it is not an AD subject.

### 5.2. Ablation Study

We evaluated some experiments as an ablation study of the proposed RBMSDL model with the MIRAID dataset to prove the efficiency of the proposed combination depicted in Table 3. We analyzed the impact of different configurations on the model’s accuracy and loss, providing insights into the effectiveness of specific components of the proposed RBMSDL model. The table includes variations in the number of multi-blocks and their corresponding accuracy rates and loss. Four different ablation studies are considered: In the first ablation study, we selected four residual-based multi-stage modules, which show 98.65% and a loss of 0.041. We also experimented with six residual-based multi-stage modules, focusing on the parameters that generated 97.97% accuracy and 0.053 loss. Utilizing seven residual-based multi-stage modules, including the parameter, produced 98.42% accuracy and 0.047 loss. Employing the five residual-based multi-stage modules with the same parameters reported 99.10% accuracy and 0.023 loss.

### 5.3. Experimental Results of the Proposed RBMSDL Model

**Figure 5:**
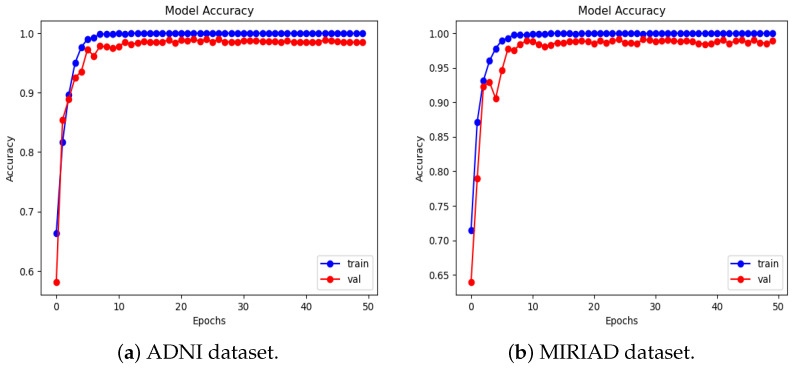
Accuracy curves of the proposed RBMSDL model with ADNI-1 and MIRIAD dataset.

**Figure 6:**
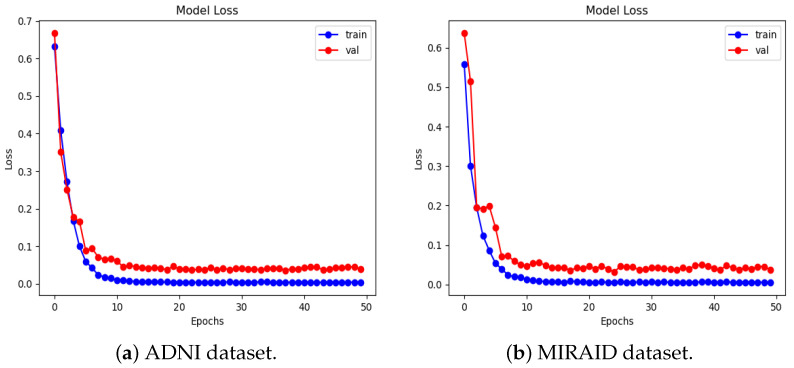
Loss curves of the proposed RBMSDL model with ADNI-1 and MIRAID dataset.

**Figure 7:**
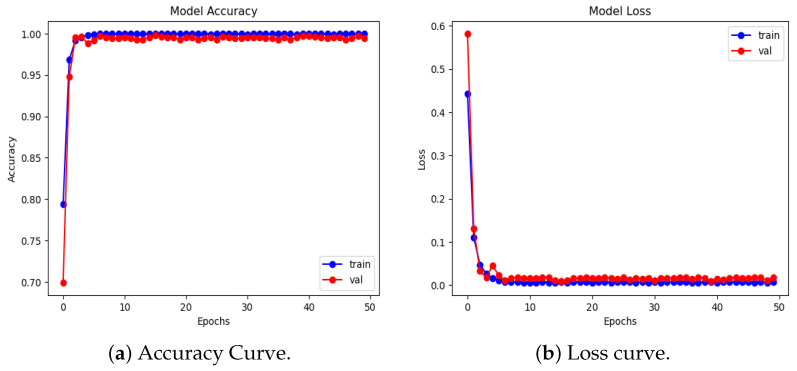
Accuracy and loss curves for our proposed RBMSDL model with OASIS dataset.

We present the experimental results of the proposed RBMSDL model for the binary classification. We evaluated the performance of our proposed RBMSDL model for three classifiers (SVM, RF, and SoftMax) with the ADNI1: Complete 1Yr 1.5T, MIRIAD, and OASIS datasets in terms of overall metrics. Table 4 tabulates the concise summary of the classification results based on adopted performance metrics. With accuracy, F1-scores, precision, and recall of 99.47%, 99.89%, 99.47%, and 99.47%, respectively, for the SoftMax classifier using the ADNI-1 dataset and 85.85%, 85.50%, 86.00%, and 86.00%, respectively, for SVM classifier using ADNI-1 dataset and 96.45%, 97.00%, 97.50% and 96.50% respectively, for RF classifier using ADNI-1 dataset, the proposed model proves itself as the best model among both ML and DL models. Similarly, using the MIRIAD dataset and achieving high accuracy, F1-scores, precision, and recall are 99.10%, 99.80%, 99.10%, and 99.10%, respectively, for SoftMax classifier and 85.18%, 85.00%, 85.50% and 85.00%, respectively, for the SVM classifier, and 94.27%, 94.50%, 94.50%, and 94.50%, respectively, for the RF classifier. The comparison results between the performances of different methods with different classifiers are summarized in Table 4 and Table 5. These outcomes suggest that integration image transformation as a feature-extraction technique within the DL framework can significantly improve accuracy and reduce training time duration. In addition, we evaluate the model based on training and validation accuracy curves and categorical cross-entropy (loss) curves, which are depicted in Figure 5, Figure 6, and Figure 7, respectively. Figure 5, Figure 6, and Figure 7 show the efficacy and the performance of the proposed RBMSDL model during training and validation with the ADNI-1, MIRAID, and Kaggle OASIS version datasets.

### 5.4. Experimental Results and Comparison for the ADNI1: Complete 1Yr 1.5T Dataset

**Figure 8:**
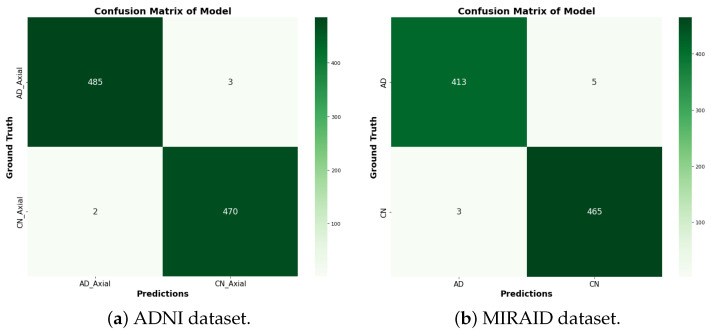
Confusion matrix of the proposed RBMSDL model with ADNI and MIRAID dataset.

Table 4 demonstrates the comparison of the proposed RBMSDL model with the existing models for the ADNI-1 dataset. The existing models were trained on binary and multi-class classification on the same datasets used in this study. As mentioned in the previous section, the proposed RBMSDL model achieved remarkable accuracy, F1-score, precision, and recall on the ADNI-1 and MIRAID datasets with three different classifiers. We compared the proposed RBMSDL model with other recent models such as TriAD [59], VGG-TSwinformer [47], Multimodel [9], Transfer learning [41], VGG 16 - VGG 19 [39], ResNet50 [24] and CNN [48]. All these existing models utilized the ADNI-1 dataset and achieved the performance as shown in Table 4. The most comparable model is Basaia et al. [58], which reported high accuracy using deep learning neural networks, but this method is inefficient in terms of computation. Venugopalan et al. [9] suggested a multi-model DL-based approach using the ADNI-1 dataset for early AD diagnosis. They used a stacked denoising auto-encoder for feature extraction from genetic and clinical data and 3D CNNs for image data. In addition, they used three classifiers (SVM, RF, SoftMax) and reported accuracy of 82.00%, 81.00%, and 86.00%, respectively. Mehmood et al. [41] used the ADNI dataset to implement layer-wise transfer learning with a VGG pre-trained model and reported an accuracy of 98.73% for distinguishing between AD and CN. Mercaldo et al. [59] same dataset used and employed a deep learning model named TriAD, for AD classification, reported an accuracy of 95.00%. Carcagni et al. [48] proposed three pre-train deep CNN models such as ResNet, DenseNet, EfficientNet, and two transformer-based models for early AD diagnosis. They used the ADNI dataset to train the models and achieved a maximum accuracy of 77.10%. Antony et al. [39] perform the AD classification task using the two pre-train models such as VGG 16 and VGG 19 [39] and reported accuracy of 81.00% and 84.00% respectively. Alsaeed et al. [24] suggested a pre-trained CNN DL-based model, ResNet50, for feature extraction for AD classification using MRI images. They implemented the models ResNet50+ SoftMax, ResNet50+SVM, and ResNet50+RF and reported accuracies of 99.00%, 92.00%, and 85.70%, respectively. Besides the existing models, our proposed RBMSDL model demonstrates the performance with three classifiers, SVM, RF, and SoftMax, and achieved high accuracy of 85.85%, 96.45%, and 99.47%, respectively. From the results depicted in Table 4, it can be seen that our proposed RBMSDL model achieved good performance in terms of accuracy, F1-score, precision, and recall as compared to other models. Figure 8a shows the confusion matrix with the SoftMax classifier on the ADNI1: Complete 1Yr 1.5T dataset while Figure 5a and Figure 6a shows the accuracy curve and loss curve respectively, which demonstrated that the proposed RBMSDL model achieved the best performance. As we can see, the ROC curve performances of the proposed RBMSDL model reach near to one, demonstrating excellent classification capability.

### 5.5. Experimental Results and Comparison for the MIRIAD Dataset

**Figure 9:**
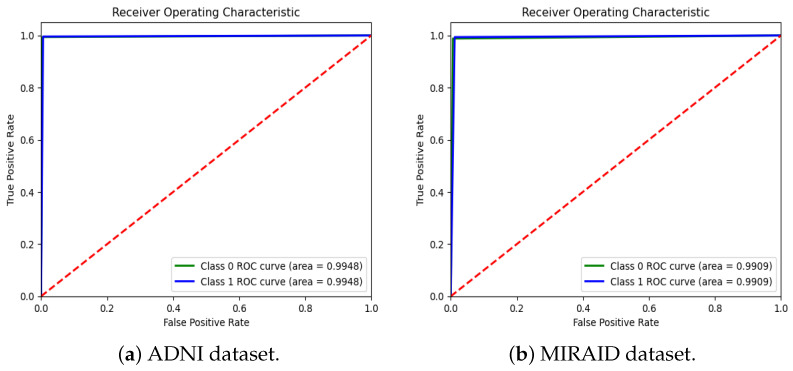
ROC curves of the proposed RBMSDL model. The diagonal (red) line indicates random chance, representing a classifier that has no discriminative power between the positive and negative classes.

We compare our proposed RBMSDL model with the existing model [24] in terms of all metrics including accuracy, F1-score, precision, and recall. As depicted in Table 5, the RBMSDL model achieved outstanding performance with three classifiers for AD binary classification. Adhaileh et al. [24] suggest a pre-trained model ResNet50 with SoftMax, SVM, and RF using the same dataset. They used the ResNet50 model to extract the features from the brain MRI images. They reported the accuracy with three classifiers (SoftMax, SVM, and RF): 96.00%, 90.00%, and 84.80%, respectively. Despite this, our proposed RBMSDL model achieved the best accuracy with three classifiers (SoftMax, SVM, and RF): 99.10%, 85.18%, and 94.27, respectively. Figure 6b and Figure 8b show the performance of our proposed RBMSDL model based on the accuracy vs. loss curves and confusion matrix respectively. Finally, it can be seen in Figure 9b that the ROC curve performance of the proposed RBMSDL model reached 99.10%.

### 5.6. Experimental Results and Comparison for the OASIS Dataset

**Figure 10:**
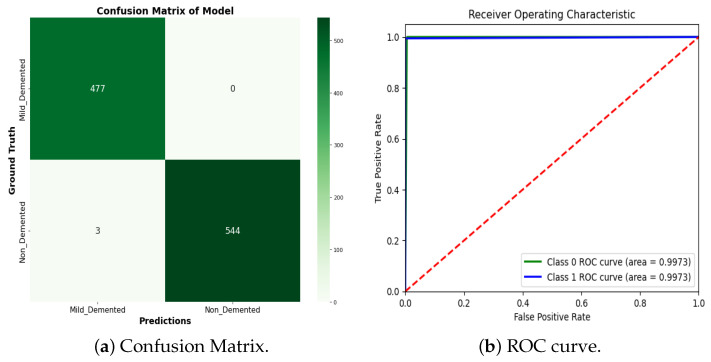
Confusion matrix and ROC curve of the proposed RBMSDL model with the OASIS dataset. The diagonal (red) line indicates random chance, representing a classifier that has no discriminative power between the positive and negative classes.

We compare our proposed RBMSDL model with the existing models using the OASIS dataset for AD binary class classification, as shown in Table 6. Specifically, the proposed RBMSDL model is compared to models such as deep ensemble [27], CNN-RFC [60], Hybrid model [61], and Multi-ML model [62] to determine the binary AD-classification task. In the existing methods we investigated, most of the comparable accuracy in Loddo et al. [27] presented a deep ensemble strategy using the same dataset for AD diagnosis and reported an accuracy of 98.51%. Similarly, Paleczny et al. [60] present two pre-trained models, Low Power Random Forest and CNN models, for AD classification using the Kaggle OASIS version dataset and achieved 95.00% accuracy. Baglat et al. [62] developed the multi-machine learning-based approaches SVM, RF, and decision tree using the OASIS dataset and reported accuracy 81.57%, 86.84%, and 81.57%, respectively. Mohammed et al. [61] developed two hybrid CNN models, AlexNet+SVM and ResNet-50+SVM, and reported an accuracy of 94.80%. The main problem with these [27,61] approaches is not only their lack of accuracy but also their computational inefficiency. To overcome these challenges, we proposed a CNN with a residual-based network to enhance the model’s efficiency and achieved good performance accuracy with three classifiers (SVM, RF, SoftMax) 91.99%, 98.92, 99.70% and more computationally efficient. Figure 7 shows the accuracy and loss curves during the training and validation duration of the proposed model. Figure 10a shows confusion matrix results, demonstrating the proposed model achieves remarkable performance for binary class classification. In contrast, Figure 10b shows the ROC curves of the proposed model for each class, i.e., AD and CN. The proposed model ROC curve is closely similar to real curves, demonstrating how the network successfully learned the pattern necessary for discriminating between the two classes.

### 5.7. Model Parameters and Loss

We compare the required number of parameters and loss value of the proposed RBMSDL model with the existing AD-recognition models as depicted in Table 7. Specifically, ADD-Net [63], Demnet [22], AlexNet [36], VGG16 [64], and VGG16+transfer learning [26] required larger numbers of parameters during training and validation. This demonstrates that our proposed RBMSDL model required fewer parameters, 155,506, during training and validation and a loss value of 0.0200, which is lower than the SOTA models.

### 5.8. Impact on Clinician Work and Discussion

This study aims to develop a system specifically designed for the AD stage utilizing MRI images to enhance diagnostic accuracy within medical centers. The first stage of the proposed RBMSDL model is the preprocessing step, as mentioned above. In the next step, we apply the proposed CNN with the residual network model to extract the effective features from the MRI images. We used multi-stage deep learning with a skip connection-based model to recognize Alzheimer’s disease. Afterward, we used three classifiers, SoftMax, SVM, and RF, to investigate the performance of the three classifiers their performance across all metrics, including accuracy, F1-score, precision, recall, number of parameters, and loss value during training and validation of our model. Because of the hierarchical effect feature, our model achieves the best performance accuracy compared to the existing system for three datasets shown in a SOTA comparison table. Besides the comparison table, we also showed the required number of parameters of the proposed model, which can also define the computational complexity.

Additionally, the proposed RBMSDL model, which showed excellence in optimizing AD diagnosis, can be adapted for other medical imaging applications such as detecting neurological disorders like Parkinson’s disease, brain tumors, and detection of various cancer diseases like lung, breast, and cardiovascular diseases. Adaptations needed include training on specific datasets relevant to each condition, adjusting the proper preprocessing techniques to emphasize pertinent features, and fine-tuning the hyperparameters for optimal performance. Moreover, validating the proposed RBMSDL model with appropriate metrics and clinical trials is important to ensure its efficacy and reliability in different medical settings.

## 6. Conclusions

Our study addresses the pressing need for effective Alzheimer’s disease (AD)-detection systems amidst the growing global health burden posed by AD. We have demonstrated significant improvements in AD-detection accuracy by leveraging advancements in deep learning techniques, particularly by introducing a novel multi-stage deep neural network architecture based on residual functions. Our model achieved the goal by generating good performance accuracy of 99.47% on the ADNI1: Complete 1Yr 1.5T dataset, 99.10% on the MIRAID dataset, and 99.70% on the OASIS dataset. The SOTA comparison table shows that our model outperformed existing systems in terms of the binary classification tasks. Systematic feature enhancement by mitigating overfitting with the suitable integration of the deep learning-based layer, including batch normalization, dropout, and fully connected layers, led to achieving robust performance across multiple benchmark datasets. While our model demonstrates exceptional performance, it may face difficulties in achieving high-performance accuracy because of the dataset sample imbalance-related issues. In the future, we are planning to explore the integration of complementary modalities and larger-scale clinical validations to consolidate and extend the impact of our proposed model in advancing AD detection and patient care besides real-world deployment.

## Figures and Tables

**Table 1:** Brief summary of the existing models.

Technique	Year	Dataset	Image Type	No. of Images	Classification	Accuracy (%)
CNN [35]	2021	ADNI	MRI	211,655	AD vs. CN	95.60
Transfer learning [41]	2021	ADNI	MRI	-	AD vs. CN	98.73
Neural Network [45]	2021	Kaggle	MRI	6400	AD vs. CN	89.84
CNN, AlexNet, GoogLeNet [43]	2021	OASIS	MRI	-	AD vs. CN	78.20, 91.40, 93.02
AlexNet, ResNet50 [36]	2022	Kaggle	MRI	1279	AD vs. MCI vs. CN	94.53, 58.07
VGG16+AOA [46]	2022	ADNI	MRI	819	AD vs. MCI vs. CN	92.34
3DCN [5]	2022	ADNI	MRI+PET	370	AD vs. NC	93.21
RS-SVM [32]	2022	ADNI	fMRI	1426	AD vs. NC	91.00
VGG16 and VGG19 [39]	2022	ADNI	MRI	780	AD vs. CN	81.00 and 84.00
ResNet50+SofMax [24]						
ResNet50+SVM [24]						
ResNet50+RF [24]	2022	ADNI	MRI	741	AD vs. CN	99.00, 92.00, 85.70
LSTM [31]	2022	ADNI	MRI	1371	AD vs. MCI vs. NC	93.87
EfficentNetB0 [44]						
EfficentNetB1 [44]						
EffcientNetB2 [44]	2022	ADNI	MRI	2182	AD vs. MCI vs. NC	93.02, 92.98, 97.28
VGG-TSwinformer [47]	2023	ADNI	MRI	-	AD vs. CN	77.20
DBN [37]	2023	ADNI	MRI	361	HC vs. AD	98.62
FDCT-WR [38]	2023	Kaggle	MRI	6400	AD vs. CN	98.71
CNN [48]	2023	ADNI	MRI	1171	AD vs. CN	77.10
Deep residual auto-encoder [49]	2023	ADNI	AD vs. CN	6400	-	98.97
VGG16+tranfer learning [26]	2024	Kaggle	MRI	6400	AD vs. NC	97.44

**Table 2:** Description of the datasets.

Dataset	Class 1	Class 2	No. of Images Class 1	No. of Images Class 2
OASIS	Non-Demented	very mild demented	3200	3200
ADNI1	AD	CN	3000	3000
MIRIAD	AD	CN	2783	2783

**Table 3:** Ablation study of the proposed RBMSDL model with MIRAID dataset.

Ablation	Number of Multi Stage Block	Loss	Accuracy
Ablation 1	4	0.041	98.65%
Ablation 2	6	0.053	97.97%
Ablation 3	7	0.047	98.42%
Ablation 4	5	0.023	99.10%

**Table 4:** The experimental evaluation results of the proposed model with SOTA algorithms using ADNI-1 dataset.

Methods	Dataset	Classifier	Recall [%]	Precision [%]	F1 Score [%]	Accuracy [%]
VGG-TSwinformer [47]	ADNI	SoftMax	79.97	-	-	77.20
Multimodel [9]	ADNI	SoftMax	85.44	80.46	88.42	86.00
Multimodel [9]	ADNI	RF	80.42	80.41	80.41	81.00
Multimodel [9]	ADNI	SVM	81.42	82.42	80.41	82.00
Deep neural network [58]	ADNI	SoftMax	98.90	-	-	99.2
Transfer learning [41]	ADNI	SoftMax	98.19	-	-	98.73
VGG16 and VGG19 [39]	ADNI	SoftMax	-	-	-	81.00 and 84.00
TriAD [59]	ADNI	SoftMax	-	-	-	95.00
ResNet50 [24]	ADNI	SoftMax	99.00	-	-	99.00
ResNet50 [24]	ADNI	SVM	87.00	-	-	92.00
ResNet50 [24]	ADNI	RF	79.00	-	-	85.70
CNN [48]	ADNI	SoftMax	-	-	-	77.10
RBMSDL	ADNI-1	RF	96.50	97.50	97.00	96.45
RBMSDL	ADNI-1	SVM	86.00	86.00	85.50	85.85
RBMSDL	ADNI-1	SoftMax	99.47	99.47	99.89	99.47

**Table 5:** The experimental evaluation results of the proposed model with SOTA algorithms using the MIRIAD dataset.

Methods	Dataset	Classifier	Recall [%]	Precision [%]	F1-Score [%]	Accuracy [%]
ResNet50 [24]	MIRIAD	SoftMax	96.00	-	97.00	96.00
ResNet50 [24]	MIRIAD	SVM	87.00	-	87.00	90.00
ResNet50 [24]	MIRIAD	RF	73.00	-	79.00	84.80
RBMSDL	MIRIAD	SVM	85.00	85.50	85.00	85.18
RBMSDL	MIRIAD	RF	94.50	94.50	94.50	94.27
RBMSDL	MIRIAD	SoftMax	99.10	99.10	99.80	99.10

**Table 6:** The experimental evaluation results of the proposed model with SOTA algorithms using OASIS dataset.

Methods	Dataset	Classifier	Recall (%)	Precision (%)	F1-Score (%)	Accuracy (%)
Multi-ML model [62]	OASIS	RF	80.00	-	-	86.84
Multi-ML model [62]	OASIS	SVM	70.00	-	-	81.57
Multi-ML model [62]	OASIS	Decsion tree	65.00	-	-	81.57
CNN RFC [60]	OASIS	RF	-	-	94.00	95.00
Hybrid Model [61]	OASIS	RF	98.00	93.00	96.00	94.00
Deep-Ensemble [27]	OASIS	Ensemble classifier	97.57	-	97.85	98.51
RBMSDL	OASIS	SVM	92.00	92.00	92.00	91.99
RBMSDL	OASIS	RF	98.50	98.50	98.00	98.92
RBMSDL	OASIS	SoftMax	99.70	99.70	99.80	99.70

**Table 7:** Computational complexity with state-of-the-art models.

Methods	Numbers of Prameters	Model Loss
ADD-Net [63]	(4,532,628 )	0.0549
Demnet [22]	(2,335,908)	-
AlexNet [36]	(60,000,000)	0.6502
VGG16 [64]	(138,627,867)	0.7600
VGG16+transfer learning [26]	(138,627,867)	0.0673
RBMSDL model	(155,506)	0.0200

## Data Availability

Data is available here: https://adni.loni.usc.edu/ and https://www.ucl.ac.uk/drc/research-clinical-trials/minimal-interval-resonance-imaging-alzheimers-disease-miriad.

## Abbreviations

ADNI	Alzheimer’s Disease Neuroimaging Initiative
SVM	Support Vector Machine
RF	Random Forest
ML	Machine learning
KNN	K-nearest-neighbor
CNN	Convolutional Neural Network
DL	Deep learning
MIRIAD	Minimal Interval Resonance Imaging in Alzheimer’s Disease
SOTA	State of the Art
RBMSDL	Residual-Based Multi-Stage Deep Learning
CLs	Convolutional Layers
DNN	Deep Neural Network
DNNs	Deep Neural Networks
CN	Control Normal
